# Tyrosine Phosphorylation within the Intrinsically Disordered Cytosolic Domains of the B-Cell Receptor: An NMR-Based Structural Analysis

**DOI:** 10.1371/journal.pone.0096199

**Published:** 2014-04-25

**Authors:** Joakim Rosenlöw, Linnéa Isaksson, Maxim Mayzel, Johan Lengqvist, Vladislav Y. Orekhov

**Affiliations:** 1 The Swedish NMR Centre, University of Gothenburg, Gothenburg, Sweden; 2 Proteomics Core Facility at Sahlgrenska Academy, University of Gothenburg, Gothenburg, Sweden; MRC National Institute for Medical Research, United Kingdom

## Abstract

Intrinsically disordered proteins are found extensively in cell signaling pathways where they often are targets of posttranslational modifications e.g. phosphorylation. Such modifications can sometimes induce or disrupt secondary structure elements present in the modified protein. CD79a and CD79b are membrane-spanning, signal-transducing components of the B-cell receptor. The cytosolic domains of these proteins are intrinsically disordered and each has an immunoreceptor tyrosine-based activation motif (ITAM). When an antigen binds to the receptor, conserved tyrosines located in the ITAMs are phosphorylated which initiate further downstream signaling. Here we use NMR spectroscopy to examine the secondary structure propensity of the cytosolic domains of CD79a and CD79b *in vitro* before and after phosphorylation. The phosphorylation patterns are identified through analysis of changes of backbone chemical shifts found for the affected tyrosines and neighboring residues. The number of the phosphorylated sites is confirmed by mass spectrometry. The secondary structure propensities are calculated using the method of intrinsic referencing, where the reference random coil chemical shifts are measured for the same protein under denaturing conditions. Our analysis revealed that CD79a and CD79b both have an overall propensity for α-helical structure that is greatest in the C-terminal region of the ITAM. Phosphorylation of CD79a caused a decrease in helical propensity in the C-terminal ITAM region. For CD79b, the opposite was observed and phosphorylation resulted in an increase of helical propensity in the C-terminal part.

## Introduction

The cytosolic domains of CD79a and CD79b are relatively short intrinsically disordered proteins (IDPs). Regions in IDPs often demonstrate transient secondary structure formation and different segments of a sequence can display a varying degree of secondary structure propensity [1]. These regions, demonstrating local structure formation, are usually involved in protein interactions [2], [3]. It is known that IDPs frequently display promiscuity and can have multiple interaction partners [4]. Adaption of IDPs to these often structurally dissimilar partners can be achieved through a process of coupled folding and binding [5]. NMR spectroscopy has proven to be one of the most informative scientific tools to study IDPs [6] and new NMR based methods are continually being developed for this purpose. Chemical shift analysis can be used to probe IDPs for transient secondary structure [7] and thus assist in identifying sites potentially important for interactions. Posttranslational modifications (PTMs) of IDPs are common and often result in a change of the binding affinity for interaction partners [8]. NMR has been extensively used to study phosphorylation of IDPs [9]–[17]. CD79a and CD79b are components of the B-cell receptor (BCR). Jointly they form a transmembrane heterodimer that is non-covalently bound to the membrane-anchored extracellular IgM molecule thereby constituting the BCR complex. The cytosolic domains of CD79a and CD79b (for simplicity hereafter referred to in this text as CD79a and CD79b) both contain an immunoreceptor tyrosine-based activation motif (ITAM)[18], a conserved motif also found in other cytosolic signaling receptor domains, e.g. the CD3-γ, -δ, -ε and ζ-domains of the T-cell receptor (TCR)[19]. The ITAM has the consensus sequence D/EX_7_D/EX_2_YX_2_L/IX_7_YX_2_L/I carrying two conserved tyrosine residues separated by a ten-residue stretch [19], [20]. Upon binding of an antigen to the IgM molecule the ITAM tyrosines become phosphorylated by Src family kinases, an event resulting in recruitment of the spleen tyrosine kinase (SYK) [21]. SYK contains two Src homology 2 (SH2) domains and phosphorylation of the two ITAM tyrosines allows for binding of SYK to the ITAMs of CD79a and CD79b via phosphotyrosine-SH2 interactions [22]. Once bound, SYK phosphorylates several proteins in the downstream signaling pathway as well as neighboring ITAM tyrosines resulting in signal propagation and amplification [22], [23]. In addition to ITAM phosphorylation, the phosphorylation of a non-ITAM tyrosine in the C-terminus of CD79a creates a docking site for the SH2 containing adaptor protein BLNK (SLP-65)[24]. BLNK then undergoes receptor-mediated phosphorylation by SYK [25], [26], an event causing BLNK to organize the assembly and activation of a multicomponent receptor-retained signalosome responsible for triggering second messenger pathways in the B-cell [27], [28].

Here we used NMR spectroscopy and chemical shift analysis to examine the secondary structure propensity of CD79a and CD79b in their non-phosphorylated and phosphorylated states and to examine how tyrosine phosphorylation affects the secondary structure propensity. A subscript letter P is used throughout this text to indicate the phosphorylated state. CD79a and CD79b were phosphorylated *in vitro* using the Src family kinase Fyn. The phosphorylated tyrosines were identified through analysis of changes of backbone chemical shifts in the vicinity of the affected sites. Our experiments shows that in their non-phosphorylated states CD79a and CD79b have helical propensity in regions centered on, or close by the C-terminal ITAM tyrosines. The helical propensity of these regions is affected by phosphorylation. For CD79b, the helicity is increased while for CD79a a decrease in helicity is observed.

## Materials and Methods

### Production of ^15^N/^13^C Labeled Cytoplasmic Domains of CD79a and CD79b

The cytoplasmic domains of human CD79a (residues 166–226) and human CD79b (residues 181–229) were produced using an *E. coli* based cell free expression system [29]. The cell free setup and purification procedures for the peptides was performed as previously described [30].

### NMR Spectroscopy

All NMR experiments were recorded on an Agilent 600 MHz spectrometer equipped with a pulse-field gradient triple-resonance cryo-probe. The CD79a experiments were recorded at 15°C and the CD79b experiments at 10°C unless stated otherwise in the text. The chemical shift of trimethylsilyl propanoic acid (TSP) was used as an internal reference. Visualization of the spectra and manual verification of the assignments were performed using the CCPN software package [31].

### Assignment of Backbone Chemical Shifts

All backbone assignments were obtained using our IDP assignment platform [30] which utilizes non-uniformly sampled BEST-TROSY type experiments [32] combined with hyper-dimensional analysis [33], targeted acquisition [34] and statistical validation. The details of this platform will not be discussed further in this paper. Lists of assigned backbone chemical shifts obtained and used in this study for the different states of CD79a and CD79b were deposited into the Biological Magnetic Resonance Data Bank (BMRB) with the following access numbers: CD79a in non-denaturing conditions nr 19644; CD79a in denaturing conditions nr 19645; CD79a_P_ in non-denaturing conditions nr 19648; CD79a_P_ in denaturing conditions nr 19649; CD79b in non-denaturing conditions nr 19650; CD79b in denaturing conditions nr 19651; CD79b_P_ in non-denaturing conditions nr 19655; and CD79b_P_ in denaturing conditions nr 19656.

### Secondary Chemical Shift Analysis

The secondary chemical shifts Δδ = δ−δ_irc_ were obtained using the method of intrinsic referencing [35], where δ is the chemical shift obtained under non-denaturing conditions and δ_irc_ is the so-called intrinsic random coil chemical shift obtained for the same protein under denaturing conditions. The experimental setup to obtain δ and δ_irc_ values for the non-phosphorylated and the phosphorylated states of CD79a and CD79b is described below.

### δ and δ_irc_ of CD79a and CD79b

The δ shifts were obtained by performing backbone chemical shift assignment for the samples prepared by dissolving purified and lyophilized ^15^N/^13^C labeled cytoplasmic domains of CD79a and CD79b in non-denaturing buffer (20 mM sodium phosphate, 150 mM sodium chloride, 2 mM DTT, 1x Complete EDTA-free protease inhibitor cocktail (Roche), 0.02% sodium azide, 100 uM trimethylsilyl propanoic acid (TSP), 12.5% D_2_O, pH 7.0). The protein concentration, determined as described in [36], was approximately 200–300 µM. To obtain the δ_irc_ shifts, urea was added to each sample to a final concentration of 6 M and the assignment procedure was repeated under these denaturing conditions.

### δ and δ_irc_ of CD79a_P_ and CD79b_P_

Phosphorylation of the cytoplasmic domains of CD79a and CD79b was peformed *in vitro* using the Src family tyrosine kinase Fyn (Life Technologies) as described in Reference [37]. Specifically, purified and lyophilized ^15^N/^13^C labeled cytoplasmic domains of CD79a and CD79b were dissolved in non-denaturing buffer to a final concentration of 200–300 µM. MgCl_2_ and ATP were then added to a final concentration of 12 mM and 1 mM respectively. To ensure that this addition did not affect the chemical shifts of CD79a and CD79b in the non-denaturing buffer, ^15^N-HSQC experiments were recorded and compared with ^15^N-HSQC experiments recorded under identical condition without MgCl_2_ and ATP. No shift changes could be observed during this comparison (data not shown). Fyn kinase was then added to a final concentration of about 300 nM along with an additional 1 mM ATP yielding a total activity estimated to approximately 2500 U. The phosphorylation reaction was run overnight at 25°C. The backbone resonances of the CD79a_P_ and CD79b_P_ samples were then assigned. To get the δ_irc_ reference shifts, urea was added to the samples to a final concentration of 6 M and the assignment procedure was repeated under denaturing conditions. Phosphorylation was verified through observations of chemical shift changes displayed by the affected tyrosines and their neighboring residues.

### Nanoelectrospray Mass Spectrometry

Unlabeled samples of CD79a and CD79b to be analyzed by mass spectrometry were produced using the same protocol as for the isotopically labeled samples. Phosphorylation of the peptides was performed at identical conditions as described above. Following phosphorylation, the buffer was exchanged to 20 mM ammonium bicarbonate with 5% formic acid using a Zeba Spin Desalting Column 7 K MWCO (Thermo Scientific) and Amicon Ultra molecular weight cut-off spin filters 3 K MWCO (Merck Millipore) with 0.1 mM tris-2-carboxyethylphosphate (TCEP) for the CD79a sample. Prior to nanoelectrospray analysis, samples were diluted 1∶1 with acetonitrile to give a final volume of 50% acetonitrile, 2.5% formic acid and 50 µM TCEP. Samples were analyzed by static nanospray on LTQ-Orbitrap Velos mass spectrometer using Proxeon borosilicate glass emitters (Thermo Fisher Scientific). The spray voltage was 1.7–2.0 kV, collecting full MS scans in positive ion mode. 1 microscan was performed at 100 000 resolution (full-width-at-half-maximum at m/z 400) over a mass range of m/z 500–2000. The full scan mass spectra with charge state distributions of non-phosphorylated and phosphorylated CD79a and CD79b are shown in Figure S2 and Figure S3. Phosphorylated CD79b was analyzed at low and high-energy to examine potential dissociation of phosphate groups (Figure S4). Theoretical protein mass values were calculated from the amino acid sequences using the MS-isotope tool in the ProteinProspector software package (http://prospector.ucsf.edu/prospector). Experimental mass values were determined from the three most abundant charge states in each case. For estimating the relative intensity of phosphorylated species, peak-top intensities were summed for each phosphorylated peak, and the corresponding sodium adduct peak, for the three most abundant charge states. After calculating the percentage of the total intensity of each phosphorylated form for each charge state, the average abundance of each phosphorylation form (Table S1) was calculated based on the result for the three most abundant charge states.

## Results and Discussion

### Secondary Structure Propensity of CD79b and CD79a

Chemical shifts are routinely used to study the structure of folded [38] as well as intrinsically disordered proteins [7]. The deviations of chemical shifts from their anticipated random coil values can be used to examine secondary structure propensity. These deviations are known as secondary chemical shifts (Δδ) and are defined as Δδ = δ–δ_rc_ where δ is the observed chemical shift and δ_rc_ is the random coil chemical shift [39], [40]. Positive C^α^ secondary chemical shift values (Δδ^Cα^) indicate prevalence of α-helical structure, while negative values point to preference towards β-strand or extended structure [38]. For Δδ^Cβ^ the sign of the effect of the secondary structure is reversed and, thus, it is convenient to monitor the difference Δδ^Cα^–Δδ^Cβ^, which is independent of the chemical shift reference [41]. Positive Δδ^Cα^–Δδ^Cβ^ values indicate a tendency for α-helical structure, and negative values indicate a tendency to β-strand or extended structure.

From the definition of Δδ, it is clear that the outcome of secondary structure predictions based on secondary chemical shift measurements relies to a high extent on the random coil chemical shifts δ_rc_, which in turn depend on the amino acid sequence and sample conditions. The traditional approach relies on using generic δ_rc_ values determined, for example, by measuring the chemical shifts for each of the twenty amino acids in short linear peptides that are assumed to be in a random coil state [42]. This method, however, is associated with several complications [35], and is particularly problematic when dealing with modified residues, e.g. phosphorylated tyrosines, for which generic values are often lacking. In this work, we explicitly calculate the secondary chemical shifts using so called intrinsic random coil shifts (δ_irc_) [35]. Namely, the intrinsic random coil shifts (δ_irc_) are determined by measuring the chemical shifts of the target peptide under denaturing conditions.

**Figure 1:**
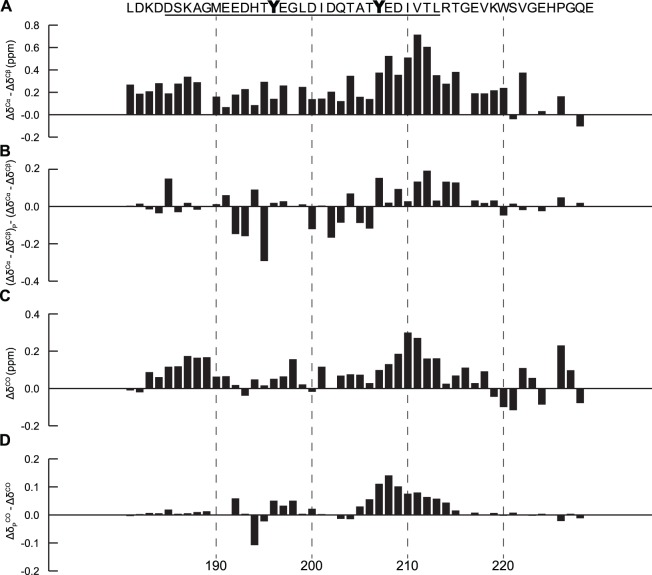
Secondary structure propensity of CD79b and the effects of phosphorylation. The ITAM region in the sequence is underlined and all shifts are plotted against their corresponding residue number (**A**) positive values of secondary chemical shifts (Δδ^Cα^–Δδ^Cβ^) indicate an overall tendency for α-helical structure with an increased propensity in the region Thr206 to Gly216. (**B**) change of secondary chemical shifts upon phosphorylation (Δδ^Cα^–Δδ^Cβ^)_P_–(Δδ^Cα^–Δδ^Cβ^). Positive values for residues Tyr207 to Gly216 indicates an increased helical content in this region following phosphorylation. (**C**) Δδ^CO^ secondary chemical shifts. The Δδ^CO^ shift pattern agrees well with the pattern of (Δδ^Cα^–Δδ^Cβ^) indicating an overall tendency for α-helical structure (**D**) change of secondary chemical shifts (Δδ_P_^CO^–Δδ^CO^). Phosphorylation increases the tendency for α-helical structure in the C-terminal part of the ITAM region.

The backbone CO secondary chemical shifts (Δδ^CO^) and the values of (Δδ^Cα^–Δδ^Cβ^) for CD79b are shown in Figure 1. There is one region in CD79b that a pattern of these parameters consistent with significant helical propensity (Figure 1A,C). This region is situated in the C-terminal region of the ITAM, stretching from Thr206 to Gly216 including the C-terminal ITAM tyrosine Tyr207. Examination of the amino acid sequence of this stretch reveals features that could be expected to promote helix formation. The transient helix starts at Tyr207 followed by the negative residues Glu208 and Asp209. It has been shown that negative amino acids are preferred at the N-terminal positions in helices due to favorable electrostatic interactions with the helix dipole [43], [44]. The helical region ends at Gly216, which is reasonable considering that glycine is a known helix breaker [45]. CD79b also had a smaller region (Asp183 to Gly189) towards the N-terminus that also displays helical propensity.

**Figure 2:**
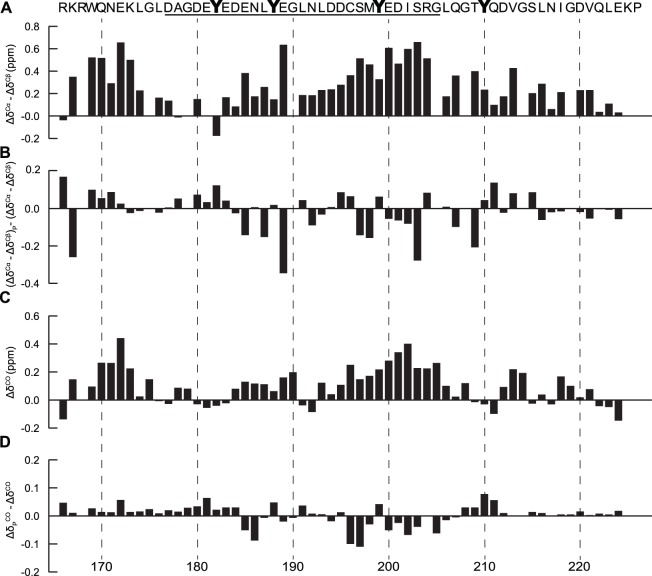
Secondary structure propensity of CD79a and the effects of phosphorylation. The ITAM region in the sequence is underlined and all shifts are plotted against their corresponding residue number (**A**) (Δδ^Cα^–Δδ^Cβ^) secondary chemical shifts. CD79a has an overall tendency for α-helical structure with an increased propensity in the regions Arg166 to Gly175 and Asp194 to Gly205. (**B**) (Δδ^Cα^–Δδ^Cβ^)_P_–(Δδ^Cα^–Δδ^Cβ^) secondary chemical shifts. Negative values in the C-terminal part of the ITAM indicate decreased helicity in this region following phosphorylation. (**C**) Δδ^CO^ secondary chemical shifts. The Δδ^CO^ shift pattern agrees well with the pattern of (Δδ^Cα^–Δδ^Cβ^) indicating an overall tendency for α-helical structure (**D**) (Δδ_P_^CO^–Δδ^CO^) secondary chemical shifts. Phosphorylation decreases the tendency for α-helical structure in the C-terminal part of the ITAM region.

The Δδ^CO^ and Δδ^Cα^–Δδ^Cβ^ values for CD79a are shown in Figure 2A,C. The secondary chemical shift patterns which are observed here are comparable to those which we have previously reported for this peptide under somewhat different sample conditions [30]. There are two regions in CD79a that display significant helical propensity. The first region is situated in the N-terminus stretching from Arg166 to Gly175. The second region is located at the C-terminal part of the ITAM stretching from Asp194 to Gly205 and includes the second of the ITAM tyrosines, Tyr199. In addition, there was also a minor region located around Tyr188 in the N-terminal part of the ITAM that displays helical tendency. It is notable that both CD79a and CD79b have a propensity for helix formation in the vicinity of, or centered on the C-terminal ITAM tyrosine.

IDPs play a key role in cell signaling [46] and it is not uncommon that interactions with their binding partners occur via a coupled folding and binding mechanism, i.e. a disorder-to-order transition takes place upon binding [4], [8]. Molecular recognition features (MoRFs) are specific regions within IDPs that are regularly involved in binding and interaction. These regions are short sequences of approximately 5 to 25 residues that, upon binding, undergo a disorder-to-order transition resulting in secondary structure formation stabilized by the binding [2], [47], [48]. Regions that adopt an α-helical structure upon disorder-to-order transitions are specified as α-MoRFs. Being short helical stretches in longer disordered regions suggests that the C-terminal ITAM regions in CD79a and CD79b are α-MoRFs and that binding to a specific interaction partner or to the cell membrane could stabilize and potentially increase the helical propensity observed in these regions. In fact, our previously published data shows that the helical C-terminal ITAM region in CD79a becomes drastically more helical in the presence of the membrane-mimetic solvent TFE [30]. Similar behavior has been observed for other α-MoRFs like a central region in myelin basic protein (MBP) [49].

Different regions in B- and T-cell receptor ITAMs have previously been observed to become helical upon interaction. A study by Gaul *et al* showed that a 12-residue peptide derived from the ITAM region of CD79a binds to the Src-kinase Lyn in an irregular helix-like conformation [50]. Futterer *et al* showed that a small region located between the two tyrosines of a dually phosphorylated ITAM peptide derived from the CD3ε chain of the T-cell receptor became helical when interacting with two SH2 domains of the kinase Syk [51]. Further, the cytosolic domain of CD3ε (CD3ε_CD_) also contains an ITAM region that becomes phosphorylated upon activation. A study by Xu *et al* has shown that in its non-phosphorylated state, CD3ε_CD_ is bound to the plasma membrane [52]. An NMR structure of CD3ε_CD_ bound to bicelles presented in the same study showed that in the bound form, segments of the CD3ε_CD_ ITAM that were inserted into the lipid bilayer were structured with helical turns surrounding the two tyrosines. Especially the region surrounding the C-terminal ITAM tyrosine was helical when interacting with the membrane. It should be noted, however, that relevance of the helical conformation for the CD79a and CD79b ITAM regions in the context of membrane binding is doubtful, since there is evidence that neither the cytoplasmic regions of CD79a nor CD79b interact with the cell membrane [53], [54].

Considering these examples, the overall α-helical propensity of CD79a and CD79b is not unexpected. However, this tendency for α-helical structure indicated by the secondary chemical shifts does not exclude the presence of other secondary structure species in solution. Since the presence of helical and β/extended structures have opposite effects on observed secondary chemical shifts, the only definite conclusion that can be drawn from our secondary chemical shift data is that, in solution, the residual helical structure has higher occupancy in comparison to the alternative conformations. Neither can we rule out the possibility of onset of non-helical structures in CD79a and CD79b upon interactions with their binding partners. It has previously been demonstrated that upon interaction with SH2 domains, ITAM residues in the vicinity of the phosphorylated tyrosines adopt an extended structure [51]. As mentioned, it is common for IDPs to have several functional conformations and adjust their structure to specific binding partners via conformational selection or coupled folding and binding [5]. In the following paragraphs we focus on the effect of phosphorylation on the observed helical propensity of CD79a and CD79b.

### Phosphorylation of CD79a and CD79b

*In vivo* the ITAMs located in the cytoplasmic domains of CD79a and CD79b are phosphorylated by members of the Src-family kinases and the SYK kinase [21]. In this study we used a recombinant version of the Src-family member Fyn to perform *in vitro* phosphorylation of ^15^N/^13^C labeled samples of CD79a and CD79b. As has been previously noted [16] and was also observed in this study (data not shown), the aromatic side-chain ^1^H-^13^C resonances of solvent exposed protein tyrosine residues show very limited chemical shift dispersion making direct determination of multiple phosphorylation states difficult. Instead, identification of phosphotyrosine positions was performed by examining backbone chemical shift changes displayed by residues surrounding the expected phosphorylation sites [16]. The differences in chemical shifts between the non-phosphorylated and the phosphorylated states of CD79a and CD79b are here defined as δ−δ_P_ where δ and δ_P_ are the chemical shifts in the non-phosphorylated and phosphorylated states respectively. If an expected phosphorylation site has a neighboring residue stretch with δ−δ_P_ values that deviate significantly from zero, this means that the site may have become phosphorylated. In contrast, a residue stretch with δ−δ_P_ values close to zero would indicate little difference between the states and would suggest an absence of phosphorylation. In general, due to possible long-range allosteric effects, observation of chemical shift perturbations of relatively distant atoms represents only circumstantial evidence for posttranslational modification at a specific site. However, for IDPs and especially under denaturing conditions, where the long-range interactions are disrupted, our method of identifying phosphorylation at specific tyrosine residues appears reasonable.

**Figure 3:**
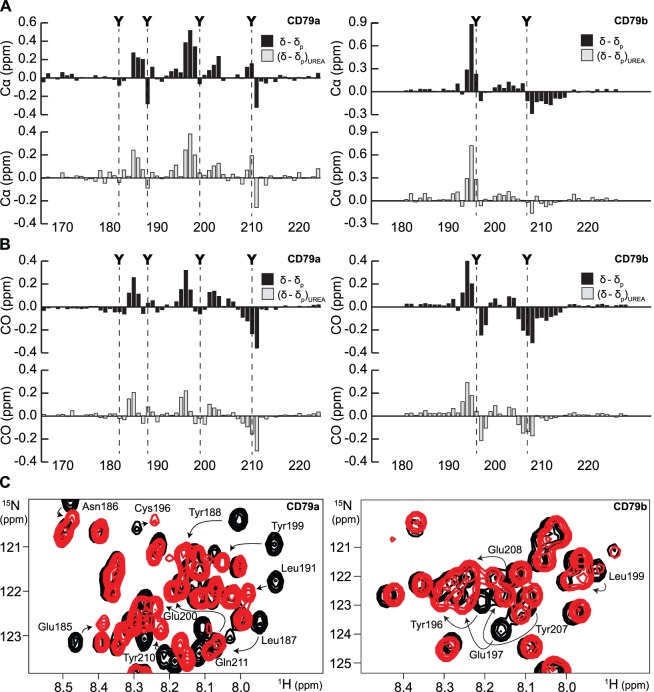
Chemical shift changes induced by tyrosine phosphorylation. (**A**) δ−δ_P_ (black bars) and (δ−δ_P_)_UREA_ (gray bars) of CD79a and CD79b calculated from Cα chemical shifts. For CD79a, significant δ−δ_P_ values can be observed surrounding Tyr188, Tyr199 and Tyr210 indicating phosphorylation of these sites. For CD79b, such values are observed surrounding Tyr196 and Tyr207. A comparison between δ−δ_P_ and (δ−δ_P_)_UREA_ reveals that a dominating part of the chemical shift changes induced by phosphorylation is still present in 6 M urea. (**B**) δ−δ_P_ (black bars) and (δ−δ_P_)_UREA_ (gray bars) of CD79a and CD79b calculated from CO chemical shifts. Analysis using CO chemical shifts results in similar patterns as Cα. (**C**) Overlays of ^1^H-^15^N-HSQC spectra of CD79a_P_ and CD79b_P_ (red) and the corresponding spectra of CD79a and CD79b (black). Phosphorylation induces changes in the amide chemical shifts of targeted tyrosines as well as surrounding residues. Phosphorylated tyrosines show a ^1^H downfield shift while the direction of the ^15^N shifts varies. Upon phosphorylation, the amide peaks of the tyrosines tend to move into already crowded areas of the spectra.

The δ−δ_P_ values of CD79a and CD79b calculated from Cα and CO chemical shifts are shown as black bars in Figure 3A and 3B. For CD79a, stretches of residues with δ−δ_P_ values deviating significantly from zero were observed on both sides of the ITAM tyrosines Tyr188 and Tyr199, and additionally on the non-ITAM tyrosine Tyr210, indicating phosphorylation of these sites. These observations are in accordance with what has previously been observed for tyrosine phosphorylation of CD79a *in vivo* [21], [24]. Based on the plots of δ−δ_P_, Tyr182 in CD79a appeared not to be extensively phosphorylated *in vitro*. Phosphorylation site predictors suggest that Fyn has a significantly lower preference for Tyr182 compared to Tyr 188, Tyr199 and Tyr210 (data not shown). Mass spectrometry analysis confirmed that the major fraction of the phosphorylated CD79a sample contains three phosphate groups (Figure S1, Table S1). For CD79b, residues with δ−δ_P_ that deviate significantly from zero were found adjacent to both ITAM tyrosines Tyr196 and Tyr207, indicating phosphorylation of these sites. Predominant phosphorylation of both ITAM tyrosines in CD79b_P_ was confirmed by mass spectrometry analysis (Figure S1, Table S1). For both CD79a and CD79b, residues with significant δ−δ_P_ values were observed as far as four residues away from the phosphorylated tyrosines. We also examined δ−δ_P_ under denaturing conditions ((δ−δ_P_)_UREA_). The (δ−δ_P_)_UREA_ values for CD79a and CD79b calculated from Cα and CO chemical shifts are shown as gray bars in Figure 3A and 3B. Comparison of (δ−δ_P_)_UREA_ with δ−δ_P_ indicates that the dominant component of the chemical shift differences resulting from phosphorylation are maintained in 6 M urea. This observation suggests that this component of δ−δ_P_ is largely contributed by the changes in random coil chemical shifts induced by the covalent attachement of the phosphate moiety.

Figure 3C shows overlays of ^1^H-^15^N-HSQC spectra of CD79a_P_ and CD79b_P_ and the corresponding spectra of CD79a and CD79b. All phosphorylated tyrosines showed a ^1^H downfield shift while the direction of the ^15^N shifts varied between the tyrosines. As has previously been observed by others [55], tyrosine phosphorylation does not appear to lead to as large perturbation of these chemical shifts as has been observed for serine and threonine phosphorylation. It is noticeable that the amide peaks of the phosphorylated tyrosines tend to move into already populated areas of the ^15^N-HSQC spectra stressing the need for experiments of higher dimensionality to resolve the peaks of individual residues when investigating tyrosine phosphorylation of IDPs.

### Effects of Tyrosine Phosphorylation on the α -helical Propensity of CD79a and CD79b

To enable a comparison between phosphorylated and non-phosphorylated CD79a and CD79b, the secondary chemical shifts of CD79a_P_ and CD79b_P_ (Δδ_P_) were calculated. It is possible to examine how phosphorylation affects the helical propensity by calculating the difference in secondary chemical shift between phosphorylated and non-phosphorylated peptides. A positive difference in either of the quantities (Δδ_P_^CO^ –Δδ^CO^) or (Δδ^α^–Δδ^β^)_P_–(Δδ^α^–Δδ^β^) indicates an increase in helical propensity and a negative difference a decrease. The (Δδ_P_^CO^ –Δδ^CO^) and (Δδ^α^–Δδ^β^)_P_–(Δδ^α^–Δδ ^β^) values of CD79b and CD79a are shown in Figure 1B,D and Figure 2B,D respectively. For CD79b, all residues starting from the C-terminal ITAM tyrosine Tyr207 to Gly216 show positive differences implying that the helical propensity of this region is increased by tyrosine phosphorylation. Previous studies have shown that when a phosphoserine is positioned at the N-terminus of a helix, this has an overall stabilizing effect on that helix [56], [57]. This stabilizing effect has been related to a favorable electrostatic interaction between the phosphoryl group and the helix dipole: it is likely that phosphorylation of Tyr207, positioned at the beginning of the helical region of CD79b, has a similar stabilizing effect. Phosphorylation of Tyr196 in CD79b did not induce a similarly large change in local helical propensity as Tyr207 although some neighboring residues showed positive values on the C-terminal side of Tyr196 and negative values on the N-terminal side.

The helical propensity of the C-terminal region centered on Tyr199 in CD79a (Asp194 to Gly205) was also affected by phosphorylation (Figure 2B,D). Here, the effect appeared to be an overall reduction of the helical propensity. It has previously been shown that a phosphoserine situated within the interior, or at the C-terminus of a helix has an overall destabilizing effect on that helix [57]. Similar destabilization has also been observed upon phosphorylation of threonine residues positioned close to the C-terminus of a helical region in the intrinsically disordered protein myelin basic protein [49]. In CD79a, Tyr199 is found close to the center of the (partly) helical region Asp194 to Gly205. Phosphorylation of this residue would thus be expected to result in destabilization of local helical structure. Phosphorylation of Tyr 188 in CD79a also resulted in a local decrease in helicity (Figure 2B,D).

Interestingly, tyrosine phosphorylation was previously reported to correlate with helix-to-coil transitions in structured systems. Aghazadeh *et al* showed that an N-terminal peptide in the Rho-guanine nucleotide exchange factor (Rho-GEF) mVav1 becomes unstructured upon tyrosine phosphorylation [58]. When in its non-phosphorylated state, the N-terminal extension forms an α-helix that autoinhibits the Dbl homology (DH) domain of mVav1 by blocking the GTPase interaction site. Phosphorylation of a tyrosine located within the helix causes release of the N-terminal inhibitory arm, exposing the interaction site and thus activating the protein. Similarly, it was shown that the largely helical N-terminal fragment of the α-chain of the pig gastric H^+^/K^+^ -ATPase was destabilized upon tyrosine phosphorylation [59]. Moreover, there are examples where binding between two proteins is regulated through phosphorylation-mediated modulation of secondary structure propensity. Phosphorylation close to the C-terminus of an α-helical region in the LD4 motif of paxillin reduces binding affinity to the FAT domain of focal adhesion kinas (FAK) through destabilization of the helix [60]. Binding between the eukaryotic translation initiation factor eIF4E and the disordered 4E-binding protein 1 (4E-BP1) is modulated by phosphorylation of a serine residue close to the C-terminal end of the binding site in 4E-BP1 [61]. In its non-phosphorylated state, a region in 4E-BP1 becomes helical upon binding to eIF4E. The phosphorylation decreased the propensity of the region to fold into the helical conformation ideal for interaction with eIF4E, thus regulating the binding. However, in the unbound form in solution, the effect of phosphorylation on the secondary structure propensity of the largely disordered 4E-BP1 was small and comparable to what is observed here for CD79a.

## Conclusions

We determined the secondary structure propensity of the cytosolic domains of CD79a and CD79b in their non-phosphorylated and phosphorylated states. Our results show that in the non-phosphorylated state, CD79a and CD79b have overall helical propensity, which is the strongest in the vicinity of the C-terminal ITAM tyrosines. Phosphorylation of CD79a and CD79b changes the helical propensity of these regions in position dependent manner similar to what has been previously observed for serine and threonine phosphorylation in IDPs.

## Supporting Information

Figure S1 **Phosphorylation state of CD79a and CD79b following** ***in vitro*** **phosphorylation.** The phosphorylated species of CD79a (**A**) and CD79b (**B**) are shown for the 5+ charge state. The number of added phosphate groups observed is indicated in the figure. Due to the high resolution of the instrument, individual charge states of the detected species are isotopically resolved. For each sample, a spectrum of phosphorylated forms is observed. The dominating forms of CD79a and CD79b contain three and two phosphate groups, respectively.
(EPS)

Figure S2 **Full scan mass spectra (** ***m/z*** **500–2000) for CD79a.** Charge state distributions observed for non-phosphorylated (**A**) and phosphorylated (**B**) CD79a. The measured mass values were in agreement with theoretical values with mass errors <3 ppm for all samples.
(EPS)

Figure S3 **Full scan mass spectra (** ***m/z*** **500–2000) for CD79b.** Charge state distributions observed for non-phosphorylated (**A**) and phosphorylated (**B**) CD79b. The measured mass values were in agreement with theoretical values with mass errors <3 ppm for all samples.
(EPS)

Figure S4 **In-source CID.** Spectra of phosphorylated CD79b analyzed under low (**A**) and high-energy (**B**). The in-source CID energy was varied from 0 to 100%. Relative intensity of the phosphorylated species was shown to be highly similar with and without in-source fragmentation (0 and 100% API source CID energy). Since there is no major shift in the relative abundance of individual phosphorylation forms, no significant dissociation of phospho-groups from the proteins occurred in the course of the nano-electrospray analysis.
(EPS)

Table S1 **The average relative abundance of phosphorylated species.** The relative abundance of different phosphorylation states of CD79a and CD79b measured in the mass spectra. The values shown here were calculated from the three most abundant charge states as described in the methods section.
(EPS)
